# The role of metabolite sensors in metabolism‐immune interaction: New targets for immune modulation

**DOI:** 10.1002/ctm2.70294

**Published:** 2025-03-30

**Authors:** Qiqing Yang, Ce Guo, Long Zhang

**Affiliations:** ^1^ Department of Radiation Oncology and the State Key Laboratory of Transvascular Implantation Devices the Second Affiliated Hospital of Zhejiang University School of Medicine Zhejiang University Hangzhou China; ^2^ MOE Laboratory of Biosystems Homeostasis and Protection and Innovation Center for Cell Signaling Network Life Sciences Institute Zhejiang University Hangzhou China; ^3^ Key Laboratory of Novel Targets and Drug Study for Neural Repair of Zhejiang Province Hangzhou City University School of Medicine Hangzhou China; ^4^ The Second Affiliated Hospital Chongqing Medical University Chongqing China

**Keywords:** immune modulation, inhibitor research and development, lactylation, metabolite sensors

## Abstract

Recent advancements in immunometabolism have highlighted the critical role of metabolite sensors in regulating immune responses. Metabolites such as lactate, succinate, itaconate, and β‐hydroxybutyrate influence immune cell function by interacting with specific sensors. These metabolites act as signaling molecules, linking cellular metabolic changes to immune responses. Lactate, a metabolite commonly produced under hypoxic conditions, has emerged as a major regulator of innate immunity. Key enzymes, including AARS1 and AARS2, function as intracellular lactate sensors, catalyzing lactylation on proteins like cGAS, which plays a central role in DNA sensing and immune activation. The lactylation of cGAS inhibits its activity, modulating immune responses by balancing inflammation and immune tolerance. Metabolite sensors, like MCT1, also contribute to immune modulation, particularly in cancer and chronic inflammatory diseases. Therapeutically, targeting these sensors offers potential for restoring immune function, especially in cancer immunotherapy. However, challenges in specificity, off‐target effects, and long‐term safety require further investigation. This article explores the emerging role of metabolite sensors in immune regulation, with a focus on lactate sensors, and outlines potential therapeutic strategies to enhance immune responses in metabolic diseases.

## Graphical abstract

- Metabolites such as lactate, succinate, and itaconate modulate immune functions through specific sensors.
- AARS1 and AARS2 are identified as key lactate sensors, regulating immune responses via lactylation of cGAS.
- Targeting metabolite sensors holds potential for improving cancer immunotherapies and treating autoimmune diseases.

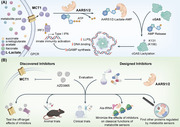

Lactate has long been regarded as a metabolic waste product. However, in diseases ranging from autoimmune disorders to viral infections, lactate has emerged as a key regulator of innate immunity. Besides lactate, many other metabolic intermediates such as succinate, itaconate and β‐hydroxybutyrate, all possess the ability to mediate modifications of histones and immune‐related proteins to epigenetically and directly regulate immune signalling pathways, respectively. Lactylation, a newly discovered post‐translational modification identified in 2019, occurs on lysine residues in numerous proteins.^1^ However, no lactyltransferase has yet been identified that can mediate lactylation on a proteomic scale. Our research has demonstrated alanyl‐tRNA synthetases AARS1 and AARS2 as intracellular lactate sensors and global lysine lactyltransferases, establishing a direct link between lactate accumulation and the inactivation of cyclic GMP‐AMP synthase (cGAS).^2^

## 1 METABOLITE SENSORS AT THE CROSSROADS OF IMMUNITY

Metabolite sensors are essential for linking cellular metabolic changes with immune regulation.^3^ Various metabolites, including lactate, succinate, itaconate, acetate, and α‐ketoglutarate, act as signalling molecules to modulate immune cell functions.^4^ These metabolic intermediates are sensed by specific proteins, primarily enzymes, which orchestrate immune responses in a finely tuned manner.

Lactate, predominantly produced under hypoxic conditions or during inflammation, is one of the most extensively studied metabolites influencing the immune system.^5^ Notably, AARS1 and AARS2 serve as pivotal lactate sensors. They catalyze the lactylation of proteins in an ATP‐dependent manner. These proteins include cGAS, a central DNA sensor in innate immunity. The lactylation of cGAS suppresses its activation, which in turn limits the production of cGAMP and the subsequent activation of the STING pathway, which is crucial for initiating antiviral and inflammatory responses. Through the modulation of cGAS activity, lactate influences the balance between immune tolerance and inflammation, particularly in conditions such as cancer and chronic inflammatory diseases. Besides AARS1 and AARS2, monocarboxylate transporter 1 (MCT1) and GPR81, also sense lactate to modulate innate immunity.

In addition to lactate, succinate serves as a danger signal by activating inflammatory responses through the GPR91 receptor.^6^ GPR43 detects acetate and other short‐chain fatty acids, enhancing the function of regulatory T cells and contributing to immune homeostasis.^7^ TET family proteins sense and utilize α‐ketoglutarate as a cofactor to mediate DNA demethylation and regulate immune gene expression.^8^ Therefore, studying the sensors of metabolites may provide novel therapeutic opportunities for regulating immune responses in diseases such as cancer, autoimmune disorders, and infections.

## 2 THERAPEUTIC IMPLICATIONS: TARGETING METABOLITE SENSORS TO RESTORE IMMUNE FUNCTION

The implications of lactylation in immune regulation are far‐reaching. In cancer, where metabolic reprogramming is a common feature, lactate accumulation in the tumour microenvironment (TME) has been shown to impair immune cell functions.^9^ Targeting lactate‐associated proteins, particularly the enzymes AARS1 and AARS2, could reverse this immunosuppressive effect and enhance the efficacy of immunotherapies. For instance, inhibiting the lactylation of cGAS could restore its DNA‐sensing ability and reactivate innate immunity, enabling the immune system to detect and eliminate tumour cells more effectively.

One promising approach involves inhibiting MCT1, the transporter responsible for lactate uptake into cells.^10^ Our research demonstrated that blocking MCT1 by AZD3965 prevents the accumulation of lactate and, consequently, inhibits the lactylation of cGAS and promotes the liquid‐liquid phase separation of cGAS. This restoration of cGAS function could have significant implications for enhancing the effectiveness of existing immunotherapies, such as checkpoint inhibitors, which are often limited by an immunosuppressive TME.^11^ Moreover, this strategy could be extended to other cancers and immune diseases where metabolic reprogramming plays a pivotal role in immune evasion.

Besides lactylation, other novel post‐translational modifications—such as succinylation, itaconation, and β‐hydroxybutyrylation—also modulate immune responses.^12^ These modifications are regulated by metabolite sensors, highlighting the potential of targeting these sensors for therapeutic intervention. Small molecules designed to target metabolite sensors could offer new treatment options for diseases in which metabolic reprogramming contributes to immune evasion, including autoimmune diseases and chronic viral infections.

## 3 CLINICAL CHALLENGES AND FUTURE PERSPECTIVES

While inhibitors or modulators of metabolite sensors may provide new therapeutic avenues for treating viral infections, cancer, and other diseases, several challenges remain in translating these findings into clinical practice.^13^ For lactate sensors, evaluating the specificity of inhibitors targeting AARS1 and AARS2 is essential to avoid off‐target effects that could disrupt normal metabolic processes.^14^ Furthermore, some of these sensors, including AARS1 and AARS2, have essential functions like mRNA translation, which need to be considered when developing inhibitors. Additionally, the long‐term effects of modulating metabolic pathways on immune function need to be thoroughly studied to assess potential risks and benefits.

In parallel, more research is also needed to map the full range of modifications mediated by metabolite sensors, such as the lactylome—the complete set of proteins that undergo lactylation in response to metabolic signals. Understanding how lactylation alters the function of these proteins will be crucial for identifying new therapeutic targets and refining treatment strategies. Finally, clinical trials will be necessary to assess the safety and efficacy of targeting metabolite sensors in human patients, particularly in combination with existing therapeutic strategies.

## AUTHOR CONTRIBUTIONS

Qiqing Yang and Ce Guo contributed equally to this work. Qiqing Yang and Ce Guo conceived and drafted the manuscript. Qiqing Yang and Ce Guo drew the figures. Long Zhang provided valuable discussion and revised the manuscript. All authors have read and approved the article.

## CONFLICT OF INTEREST STATEMENT

The authors declare no conflict of interest.

## FUNDING INFORMATION

The work mentioned in this editorial was supported by the Chinese National Natural Science Funds (31925013, U20A20393 and W2411011), a special programme from the Ministery of Science and Technology of China (2021YFA1101000 and 2024YFC2707400), a Key R&D Program of Zhejiang Province (2023C03044 and 2024C03142) and the joint project of pinnacle disciplinary group, the second affiliated hospital of chongqing medical. We thank G.Xiao and Z.Lin from the core facilities, Zhejiang University school of medicine for technical support.

## ETHICS STATEMENT

Not applicable.

## Data Availability

Not applicable.
